# Models for predicting bulinids species habitats in southwestern Nigeria

**DOI:** 10.1016/j.parepi.2022.e00256

**Published:** 2022-05-30

**Authors:** Opeyemi G. Oso, Joseph O. Sunday, Alex B. Odaibo

**Affiliations:** a Parasitology Research Unit, Department of Zoology, University of Ibadan, Ibadan, Nigeria; b Department of Zoology, Kwara State University, Malete, Nigeria

**Keywords:** Bulinid species, GIS/RS, Schistosomiasis, Nigeria

## Abstract

**Background:**

Schistosomiasis prevalence is high in southwestern Nigeria and planorbids of the genus *Bulinus* had been implicated in the transmission of the disease in the area. The knowledge of species distribution in relation to environmental variables will be auspicious in planning control strategies.

**Methods:**

Satellite imagery and geographic information system (GIS) were used to develop models for predicting the habitats suitable for bulinid species. Monthly snail sample collection was done in twenty-three randomly selected water contact sites using the standard method for a period of two years. Remotely sensed variables such as Land Surface Temperature (LST), Normalized Difference Vegetation Index (NDVI) were extracted from Landsat TM, ETM^+^; Slope and Elevation were obtained from digital elevation model (DEM) while Rainfall was retrieved from European Meteorology Research Program. These environmental factors and snail species were integrated into QGIS to predict the potential habitats of different bulinid species using an exploratory regression model.

**Results:**

The following environmental variables: flat-moderate slope (0.01–15.83), LST (21.1 °C-23.4 °C), NDVI (0.19–0.52), rainfall (> 1569.34 mm) and elevation (1–278 m) contributed to the model used in predicting habitat suitable for bulinids snail intermediate hosts. Exploratory regression models showed that LST, NDVI and slope were predictors of *Bulinus globosus* and *Bulinus jousseaumei*; elevation, LST, rainfall and slope were predictors of *Bulinus camerunensis*; rainfall, NDVI and slope were predictors of *B. senegalensis* while NDVI and slope were predictors of *Bulinus forskalii* in the area. Bulinids in the forskalii group showed clustering in middle belt and south. The predictive risk map of *B. jousseaumei* was similar to the pattern described for *B. globosus*, but with a high R-square value of 81%.

**Conclusion:**

The predictive risk models of bulinid species in this study provided a robust output for the study area which could be used as base-line for other areas in that ecological zone. It will be useful in appropriate allocation of scarces resources in the control of schistosomiasis in that environment.

## Graphical abstract

**Figure:**
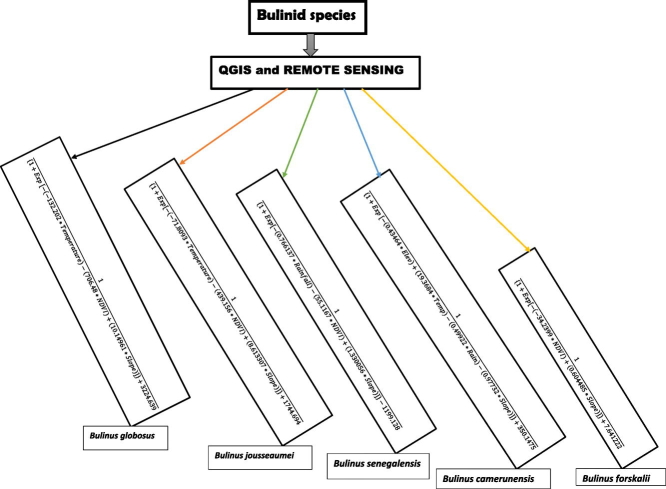

## 1 Introduction

Schistosome infection causes debilitating illnesses in millions of children and adults in different parts of the world, especially in tropical countries. Freshwater snails continue to play a significant role in the transmission of the infection. They invade freshwater bodies where they serve as intermediate hosts, transmitting several parasites (Dundee and Paine, 1977; Bogea et al., 2005). Different stages of the life cycle of schistosome parasites are completed in the snail species. These intermediate hosts inhabit a wide range of natural and man-made habitats and they are often found in irrigation canals, dams, ponds and ditches (Brown, 1994; Rollinson et al., 2003; Phiri et al., 2007). Therefore, the proliferation of these freshwater snails needs to be scientifically monitored extensively (Brown, 1994; Rollinson et al., 1998; Supian and Ikhwanuddin, 2002; Allan et al., 2020).

Studies in Southwestern Nigeria have shown that prevalence of schistosome infection among the inhabitants and snail intermediate hosts are high (Salawu and Odaibo, 2013; Akinwale et al., 2015). The mainstay of schistosome treatment in humans is praziquantel-based therapy; while snail control is almost neglected or perhaps they are considered as an accompanying strategy, most especially in high transmission areas (Chen, 2005; Allan et al., 2020). It has been observed that embarking on large–scale control of snails seems to be impracticable, however, identification of areas at high risk and application of long-term effective control measures have emerged as a possible way of interrupting schistosome transmission (Zhang et al., 2009; Hu et al., 2013).

Field epidemiology is often based on the fact that definitive host, snail intermediate hosts and their associated pathogen are associated with certain environmental factors. These environmental factors either increase the survival of snail species or inhibit them (Reisen, 2010). However, the development of geographic information system and remote sensing technology have provided a more robust way of determining environmental variables which are related to the distribution of snail intermediate host of schistosomes (Pavlovsky, 1966; Brooker and Michael, 2000).

There are about forty known generations of planorbids that are found on different continents where schistosomiasis is prevalent, in almost any freshwater lake, pool, or stream (Malek, 1985; Zhang et al., 2010). In all, there are approximately 37 recognized species of *Bulinus* species (Brown, 1994); however, the specificity of the snail–parasite interaction is such that only certain species are involved in the transmission of the parasite. The genus can be further divided into four major groups, namely, *Bulinus africanus* group, *Bulinus forskalii* group, *Bulinus reticulatus* group and *Bulinus truncatus/tropicus* complex. In each group, there are species that act as intermediate hosts of trematodes in different parts of the world (Brown, 1994). The growing interest in biodiversity and its evaluation has highlighted the importance of species identification (Ronquist and Gardenfors, 2003), but the distribution of these snails is related to availability of freshwater bodies and suitable environmental factors. Therefore, to understand the transmission dynamics of schistosome infection in relation to snail intermediate host, it is necessary to have a precise knowledge of prevailing environmental variables in time and space. Geographic Information System (GIS) and Remote Sensing (RS) have proved to be useful for epidemiological research purposes, decision making, planning, management and dissemination of information in time and space. GIS applications related to health have been introduced and used in, for example, the surveillance and monitoring of vector-borne diseases (Beck et al., 1994; Glass et al., 1995; Bernardi, 2001; Kistemann et al., 2002; Bedard and Henriques, 2002; Maantay, 2002; Bédard et al., 2003). Remote sensing and GIS have also increased their importance and utility in health-related studies (Rytkönen et al., 2001; Rytkönen et al., 2003; Berke, 2010; Bowman et al., 2010). Environmental variables such as climate, satellite sensor data, elevation, slope, land use and land cover, soil type, and other map data are overlaid on a base map of standard geographic projection and scale. This study was designed to develop environmental parameters for mapping and predicting suitable habitats for bulinid species in disease-endemic areas.

## 2 Materials and methods

Coordinates of the sampling sites were determined using a GPS (Magellan Explorist 310, MiTAC Digital Corporation, CA 95050 USA). The study was carried out in Yewa North Local Government Area (YNLGA), a local schistosome transmission site in southwestern Nigeria (latitudes 6^o^52’08”N to 7^o^25’28”N and longitudes 2^o^43’09″E to 3^o^07’13″E). It has a land size of about 200,214km^2^. It shares boundaries with Imeko-Afon local government area in the North, Yewa South Local Government Area in the South, the Republic of Benin in the West and Abeokuta North and Ewekoro local government areas in the East.

### 2.1 Data collection

Yewa North LGA has the largest landmass in Ogun State with forty-nine identified villages, each village having water contact sites. Each of the villages was visited for snail sampling before the study started. Water contact sites without snail species were excluded from the study while other water contact sites (with or without bulinid species) were included in the study. After initial pre-sample collection, a total of twenty-three water contact sites were randomly selected for snail collection and analysis. Once a month, bulinid species (*Bulinus globosus*, *Bulinus jousseaumei*, *Bulinus camarunensis*, *Bulinus senegalensis* and *Bulinus forskalii*) were collected from water contact sites using scoop net for two years. Snail identification and infection status were done using morphology and molecular methods respectively. Results of the snail identification and infection have been published elsewhere (Salawu and Odaibo, 2013; Akinwale et al., 2015).

The monthly rainfall data was obtained from the European Meteorology Research Program (http://apps.ecmwf.int). The data set has a spatial resolution of 0.7 m. The data was downscaled using the multi-dimensional tool in QGIS software. NDVI was generated using the near infra-red band and the red band. The value of the NDVI ranges from −1 to 1, values lesser than 1 show that the areas are not vegetated while vegetation condition improvement has it tends to 1. The Digital Elevation Model (DEM) of the Advanced Space-borne Thermal Emission Radiometer (ASTER) was obtained from the National Aeronautical and Space Agency (NASA). The Slope image was extracted from the Digital Elevation Model and was converted using the spatial analysis tool in the QGIS. The slope was grouped into various classes ranging from very steeply to flat. The unit of the slope was measured in percentage. The thermal band of Landsat ETM+ sensor was used to derive Land Surface Temperature of the study area. For the Landsat ETM^+^ sensor, images in the thermal band were captured twice: once in the low-gain mode (band 6 L) and once in the high-gain mode (band 6H) (Artis and Carnahan, 1982).

### 2.2 Data analysis

An exploratory regression model was used as a method to investigate all potential explanatory variables that may be important contributing environmental factors for estimating the potential habitat of snail species. Independent variables such as rainfall, slope, Normalized difference vegetation index (NDVI), and Land surface temperature (LST) were used while the different snail species serve as the dependent variables. After careful consideration of the theory and examination of the data using the exploratory regression method, one significant model presented itself as most suitable for predicting the locations of the snails. The feasibility of the modeled environmental suitability based on remotely sensed data was validated with reference to on-site observations after the model was created. The model generates the equation as shown below:

$$Y=\beta_{0}+\beta_{1}X_{1}+\beta_{2}X_{2}+\ldots \text{βnXn}+\varepsilon$$

Where, Y = Dependent variable, X = Explanatory variable e.g. environmental factors, Intercept = (β_0_), Coefficients = (β_1_…βn), Residuals = (ε).

The probability map was generated for each of the snail species with values ranging from 0 to 1.

$$P=\frac{1}{1-e^{z}}$$

Where, P = probability of occurrence, e = exponential, z = regression model obtained from the OLS (Y = β_0_+ β_1_X_1_+ β_2_X_2_ + … βnXn+ ε).

## 3 Results

Environmental factors used for building the model were extracted from the satellite imagery and digital elevation model (Table 1). The slope of the area was categorized into flat (0.01–4.75), gentle (4.76–9.23), moderate (9.24–15.83) and steeply (>15.84). Eastern areas had steeply slope while southern areas were flat. Moderate NDVI was recorded in the south while the middle belt had slightly lower NDVI values. Northern areas were higher compared to the southern areas which were lower in terms of elevation. LSTs were within the tolerance limit in the south while the north had higher values. Rainfall patterns were more in the north-west compared to south-east.

**Table 1:** Minimum and Maximum values of environmental variables.

Environmental variables	Minimum and Maximum values
Slope	0.01–67.26
NDVI	0.02–0.52
Elevation (m)	1.0–278
LST(°C)	21.1–27.7
Rainfall (mm)	1569.34-1590.02

**Fig. 1:**
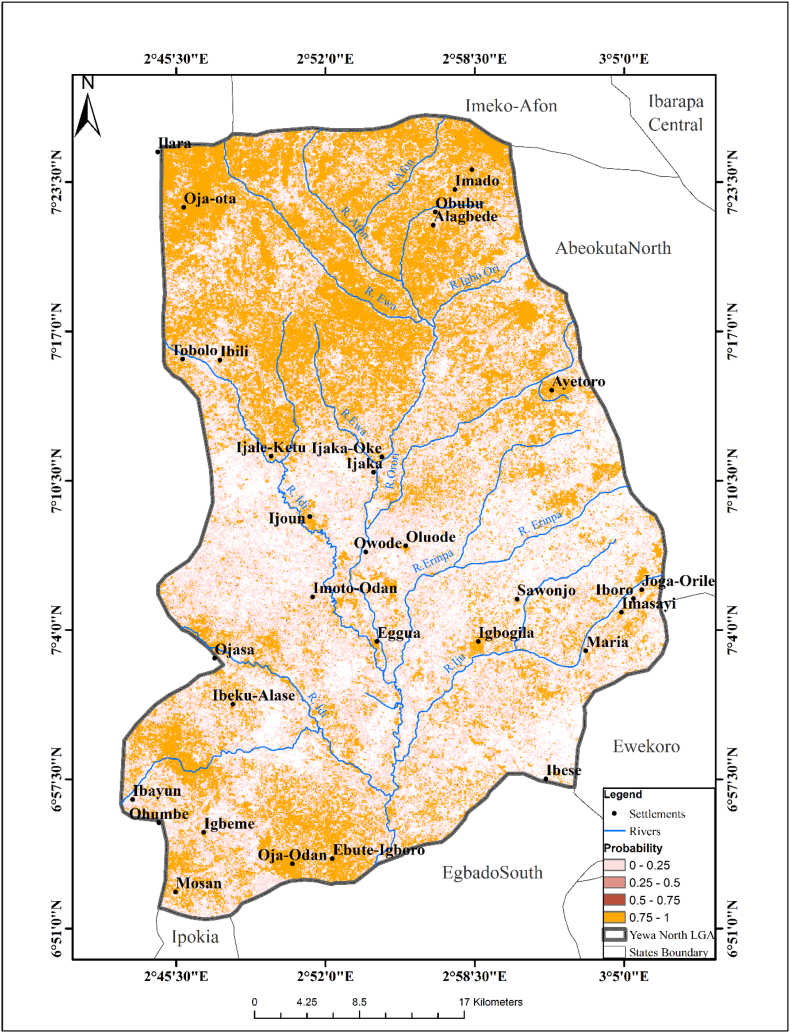
Predictive Risk Map of *Bulinus globosus* Habitat.

The predictive model showed that most areas in YNLGA were suitable for the survival of *B. globosus* except for some areas toward the middle. The exploratory regression analysis showed that temperature, NDVI and slope were the three major significant variables in predicting the geo-spatial distribution of *B. globosus* (P < 0.05). The passing model using the R square and Akaike's Information Criterion (AICC) identified Imasayi, Ijoun, Oja-Odan, Oja-Ota and Ijale- Ketu as major areas where *B. globosus* can survive. A predictive risk map of *B. globosus* habitat was created based on the final exploratory regression analysis (Fig. 1). High risk areas were mainly located in Iyalode, Imasayi, Agadda, Ijoun, Fagbire, Oja-Odan, Adelabu, Oja Ota, Apata and Ijale-Ketu while low risk areas were Ijaka, Akinsanya, Sawonjo, Alagbe, Abule-Awba and Ibese. The exploratory regression model of the probability of presence of *B. globosus* is stated below:

$\mathrm{Predictive risk model of}\;B.\mathrm{globosus}\;\mathrm{habitats}=\frac{1}{\left\{1+\mathrm{Exp}\;\left[-\left(-132.202*\mathrm{Temperature}\right)-\left(706.48*\mathrm{NDVI}\right)+\left(10.14961*\mathrm{Slope}\right)\right]\right\}+3224.639}$

**Fig. 2:**
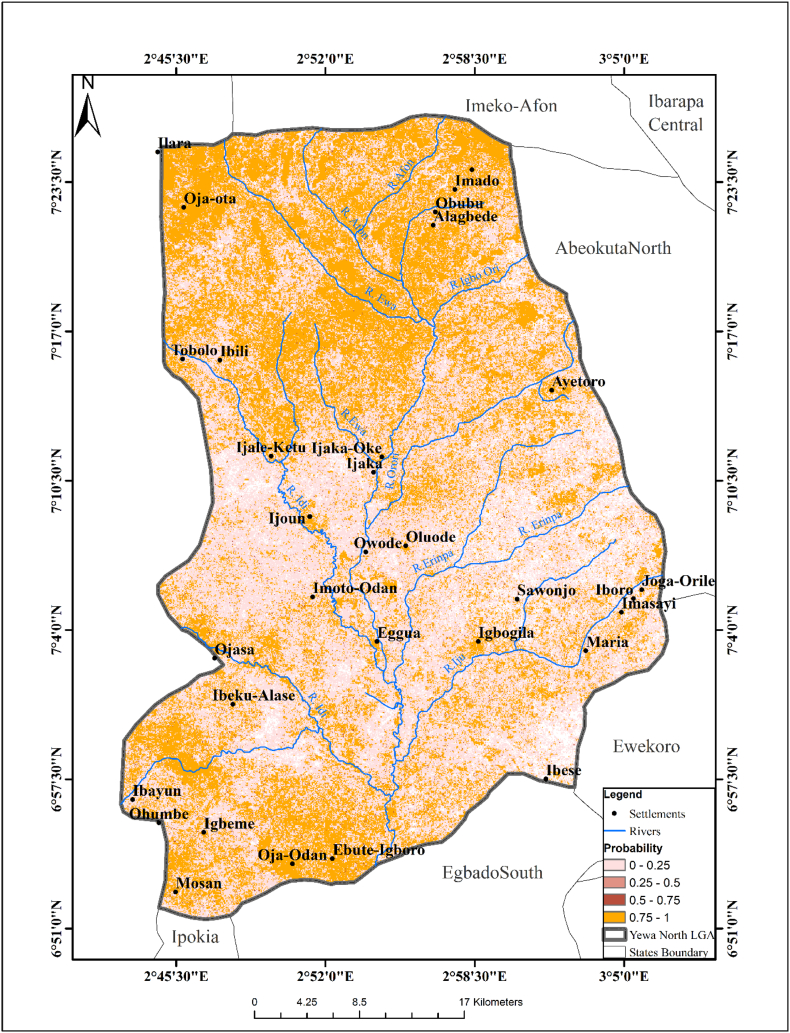
Predictive Risk Map of *Bulinus jouseaumei* Habitat.

The predictive risk map of *Bulinus jousseaumei* followed the same pattern as *B. globosus*, however, the predictive risk map of *B. jousseaumei* had a higher R square value of 81%. Temperature, NDVI and slope were the major variables used in the analysis (*P* < 0.05). A predictive risk map of the habitat was created based on the final exploratory regression analysis (Fig. 2).

The exploratory regression model of the probability of *B. jousseaumei* is stated below:

$$\text{Predictive risk of}\;B.\mathrm{jousseaumei}\;\text{habitats}=\frac{1}{\left\{1+\mathrm{Exp}\left[-\left(-71.8093*\mathrm{Temperature}\right)-\left(439.156*\mathrm{NDVI}\right)+\left(8.613307*\mathrm{Slope}\right)\right]\right\}+1744.694}$$

**Fig. 3:**
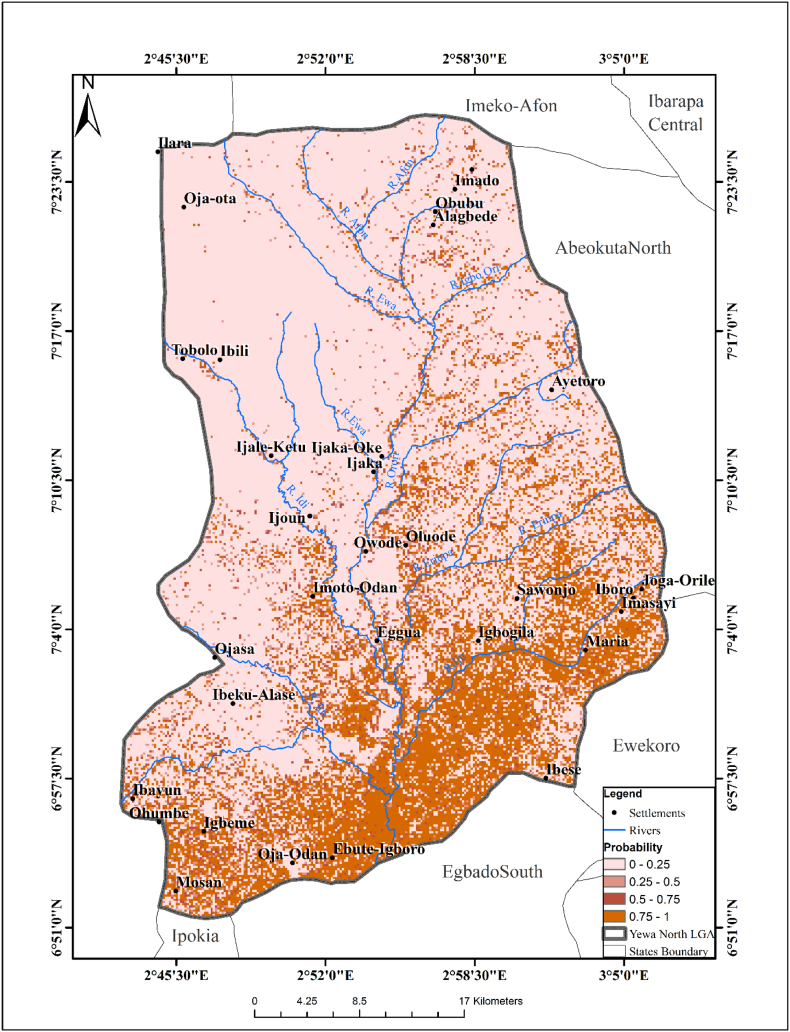
Predictive Risk Map of *Bulinus senegalensis* Habitat.

Most of the areas in Southeastern part of YNLGA were suitable for the survival of *B. senegalensis* except in some areas in the northwest. The exploratory regression analysis showed that rainfall, NDVI and slope were the major spatial variables used in the model (*P* < 0.05). Areas around Abule-Egba, Imina, Edun, Imasayi, Igbogila, Oja-Odan, Mosan, Owode, Fatolu and Ebute-Igboro were identified as suitable for the survival of *B. senegalensis*. A predictive risk map of habitats was created based on the final exploratory regression analysis (Fig. 3).

$$\text{Predictive risk of}\;B.\mathrm{senegalensis}\;\text{habitats}=\frac{1}{\left\{1+\mathrm{Exp}\left[-\left(0.766137*\mathrm{Rainfall}\right)-\left(55.1167*\mathrm{NDVI}\right)+\left(1.330056*\mathrm{Slope}\right)\right]\right\}-1199.128}$$

**Fig. 4:**
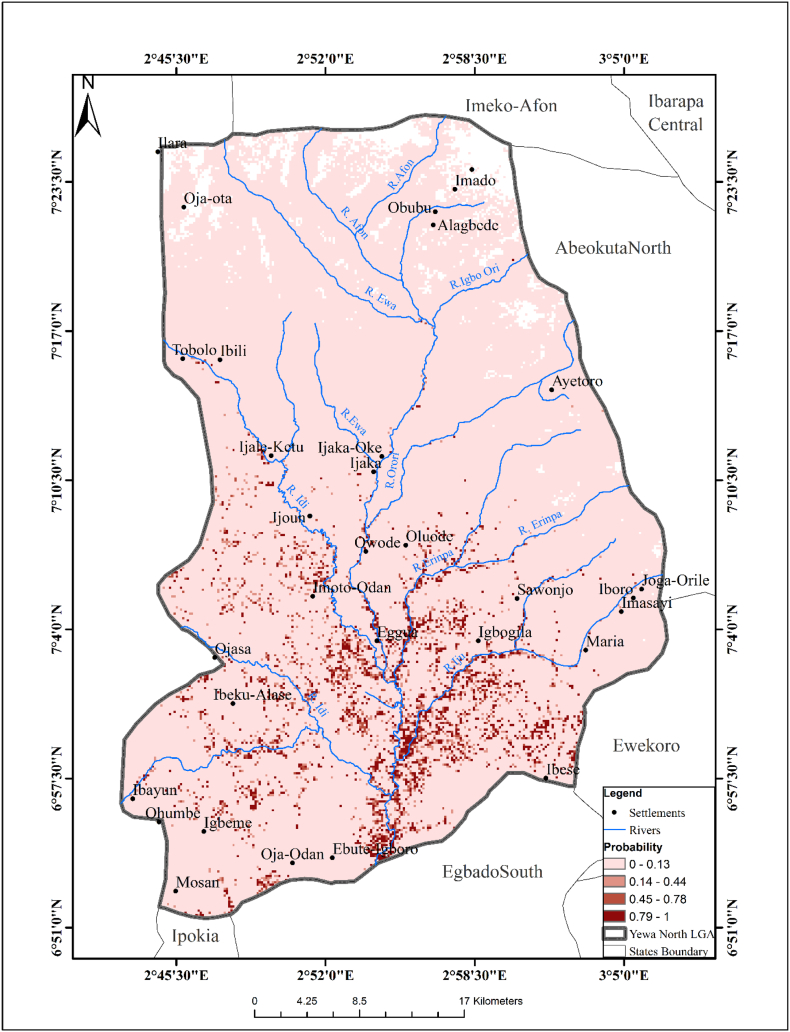
Predictive Risk Map of *Bulinus camerunensis* Habitat.

Fig. 4 showed the predictive risk map of *Bulinus camerunensis*. The northern parts of the YNLGA were not suitable for the survival of *B. camerunensis* while most areas in the south were suitable for the survival of the species. The following variables: elevation, temperature, rainfall and slope were maintained in the analysis (*P* < 0.05). The passing model using the R square (99.2%) and Akaike's Information Criterion (AICC) identified Eggua, Ijale-Ketu, Imoto-Odan, Igbogila, Agbon and some other areas with the same digital value as suitable areas where *B. camerunensis* can survive. The exploratory model of the probability of the presence of *B. camerunensis* is stated below:

$$\text{Predictive risk of}\;B.\mathrm{camerunensis}\;\text{habitats}=\frac{1}{\left\{1+\mathrm{Exp}\;\left[-\left(0.43464*\mathrm{Elev}\right)+\left(19.3684*\mathrm{Temp}\right)-\left(0.49922*\mathrm{Rain}\right)-\left(0.97732*\mathrm{Slope}\right)\right]\right\}+350.1475}$$

**Fig. 5:**
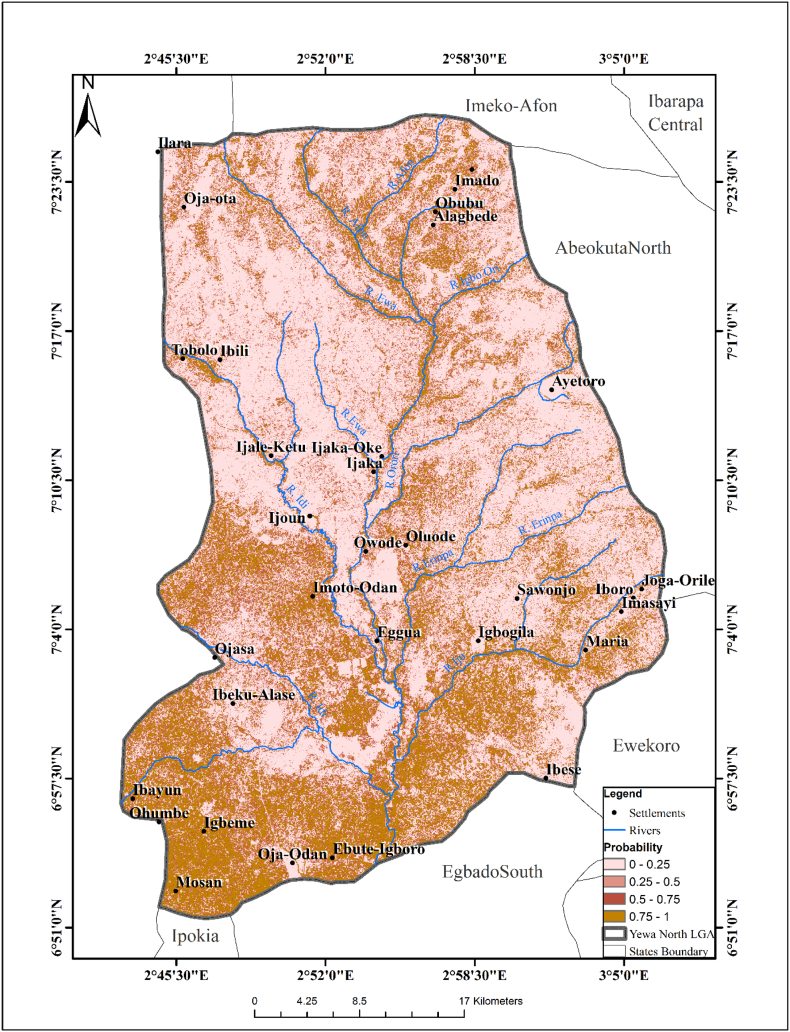
Predictive Risk Map of *Bulinus forskalii* Habitat.

The exploratory regression analysis showed that NDVI and slope were the two significant variables in predicting the geo-spatial distribution of *Bulinus forskalii* (*P* < 0.05). The passing model using the R square (82.9%) and Akaike's Information Criterion (AICC) identified Ibayun, Mosan, Ebute, Imo-Odan and Tobolo as some of the major areas where *B. forskalii* can survive. A predictive risk map of the habitat was created based on the final exploratory regression analysis (Fig. 5). The exploratory regression model of the probability of the presence of *B. forskalii* is stated below:

$$\text{Predictive risk of}\;B.\mathrm{forskalii}\;\text{habitats}=\frac{1}{\left\{1+\mathrm{Exp}\left[-\left(-34.2399*\mathrm{NDVI}\right)+\left(0.604485*\mathrm{Slope}\right)\right]\right\}+7.641222}$$

## 4 Discussion

Our study used the knowledge of GIS technology and dependent variables to create models which will be useful in monitoring the survival of snail intermediate hosts in areas where data collection could be problematic. The application of GIS and remote sensing in science-based study is of tremendous importance in disease mapping and prediction, most especially in non-sampled areas (Hay, 2000). The use of GIS/RS have been successfully used for predictions in some parts of African countries (Malone et al., 1997; Malone et al., 2001; Kabatereine et al., 2004; Raso et al., 2005). Our study found that a larger percentage of the study area had NDVI of between 0.19 and 0.52, indicating the presence of vegetation cover. The negative association observed between the NDVI and most of the bulinids was in deviance with other studies conducted in Brazil and China (Simoonga et al., 2008; Yang et al., 2009; Scholte et al., 2014), however, it was in consonance with other observations elsewhere (Clements et al., 2008; Ajakaye et al., 2017; Kulinkuna et al., 2018). NDVI is an important vegetation index and has been used in predicting the habitat of freshwater snail intermediate host of schistosomes in different ecological zones (Kristensen et al., 2001; Zhang et al., 2003; Raso et al., 2005). Besides vegetation cover, availability of humidity is an important environmental parameter for snail intermediate hosts prediction. Furthermore, an increase in anthropogenic activities in an area could result in low wetness, hence, the amount of humidity will reduce. Our study area is categorized as meso-scale, hence, a negative relationship was observed. At meso-scale, such as village survey, a moderate NDVI and high wetness could increase the survival of snail intermediate hosts while low NDVI values and low wetness could reduce the probability of suitable snail habitats (Bavia et al., 2001; Zhang et al., 2008; Yang et al., 2009). For macro-scale, such as country surveys, higher NDVI values indicate a relatively higher vegetation cover, possibly increasing the probability of potential snail intermediate host habitats (Yang et al., 2009).

The rainfall data for the study area was within limits; hence, it appeared favourable for the survival of freshwater snail intermediate host. The positive correlation between rainfall data and most of the snail intermediate hosts was in agreement with another study in Cross River State (Adie et al., 2014). Rainfall is one of the major climatic conditions that influence the distribution and abundance of snails and the rate of schistosomal development in the snail hosts (Appleton, 1978; Sturrock, 1993). In our study, the optimum LST suitable for snail intermediate host to thrive very well is between 21.1 °C and 23.4 °C, other LSTs above 23.5 °C seems to be lethal to intermediate snail hosts. The significant positive relationship between LST and some of the bulinids was in deviance with another study (Scholte et al., 2014). However, in Tanzania, no significant relationship occurred between LST, snail population and schistosome infection (Clements et al., 2008; Kulinkuna et al., 2018). Freshwater snail intermediate host of schistosomes has well-defined land surface temperature for optimal development. Land Surface Temperature is one of the determinant factors that affect the transmission dynamics of schistosome infection; it is known to influence the rate of miracidia penetration, shedding of cercaria and the length of the pre-patent infection period (Anderson et al., 1982). A study in Ethiopia showed that satellite-derived LSTs of 20-33 °C values were able to define the distribution of *Schistosoma* infection (Malone et al., 2001). However, in Uganda and other areas, by contrast, no association was observed between the prevalence of schistosomes and LST (Kabatereine et al., 2004; Ajakaye et al., 2017).

Apart from the northern part of the study area, elevation values were within the tolerance range for snail species to survive. Landscape pattern analysis can provide indications of whether an area offers suitable habitats for snail intermediate hosts to survive. Repeated analyses and inference from comparable settings might also enable the prediction of changes in the snail population resulting from ecologic transformation caused by human activities (Steinmann et al., 2006; Li et al., 2007) or deliberately targeted interventions for snail control. From our study, slope ≤ 4.75 enhances the survival of snail intermediate host while slope ≥ 15.83 may not provide a suitable habitat for snail intermediate host. The positive association that occurred between slope and most of the bulinid was in consonance with some findings in eastern Africa (Yang et al., 2009; Schur et al., 2011). Low/flat areas had a positive effect with respect to the risk of *Schistosoma* infection in China (Yang et al., 2009). In another study, inhabitants of a village situated on steeply slopes were at a lower risk of *Schistosoma* infection compared to people living in plain areas, this was due to the fact that the plain areas presented suitable habitat for bulinid snails to thrive, while steeply areas could not hold water (Steinmann et al., 2007). Water bodies found in high sloppy areas are often characterized with high water flow velocity, which does not hold the water, and the fast flow could be too fast for the intermediate host to maintain their existence in such areas. Therefore, the possibility for the freshwater snails to colonize and survive in such areas decreases as the slope increases (Zhu et al., 2015).

The following bulinids (*Bulinus senegalensis, Bulinus camerunensis* and *Bulinus forskalii*) had a higher probability of surviving in the middle belt and southern part of the study area. These three bulinids belong to the forskalii group and observation from the environmental variables which contributed to their habitat prediction, in different combination, could be as result of the tolerance level of those environmental variables (LST, rainfall, NDVI, slope and elevation). However, LST did not contribute to the building model for *B. forskalii* and *B. senegalensis*, indicating their low tolerance for high LST. The predictive risk model for *Bulinus jousseaumei* was similar to the pattern described for *B. globosus*. This could be because the two species are sympatric. The following environmental factors (LST, NDVI and slope) were part of the building model for *B. globosus* and *B. jousseaumei*. Like *B. camerunensis*, the ability of LST to form part of the building model for *B. globosus* and *B. jousseaumei*, could be as a result of the ability of the two species to adapt to high LST. In Nigeria, bulinid species in forskalii group and *B. jousseaumei* are not widely reported, however, *B. globosus* is cosmopolitan in areas where *Schistosoma haematobium* is prevalent. The ability of *B. globosus* to survive in some areas in our present study could be traced to the long term adaptation of the species to different ecological zones, however, the species were not found in higher elevated areas (>250). In conclusion, this study provides a more appropriate approach to identifying a combination of environmental variables in modeling the habitat suitable for the survival of bulinid species. Hence, our predictive risk map could serve as a guide for the effective utilization of scarce resources in the control of schistosomiasis. The predictive model in our current study showed a strong correlation with random observations from field study after the model was created. The lack of advanced satellite imagery for this study is one of our major limitations. Availability of imagery such as Geo Eye and World View will give more detailed data for better analysis.

## Author's contributions

OAB conceived the idea; OGO and JOS carried out the sampling procedure and statiscal analysis; OGO wrote the literature review and drafted the first version of the paper. All authors read, contributed and approved the paper.

## Funding

The authors acknowledge the financial support from Wellcome^Trust^ Funded Institute of Infectious Disease of Poverty (IIDP) given to OGO.

## Ethics approval and consent to participate

We obtained an approval to carry out this study from Ogun State ministry of health. We also got an approval from the village heads via thorough focus group discussion.

## Consent for publication

Not applicable.

## Declaration of Competing Interest

The authors declare that they have no competing interests.
